# Micronutrient Deficiencies in Pediatric IBD: How Often, Why, and What to Do?

**DOI:** 10.3390/nu17091425

**Published:** 2025-04-24

**Authors:** Tiziana Galeazzi, Sara Quattrini, Elena Lionetti, Simona Gatti

**Affiliations:** Department of Pediatrics, Polytechnic University of Marche, 60123 Ancona, Italy; t.galeazzi@univpm.it (T.G.); sara.quattrini29@gmail.com (S.Q.); m.e.lionetti@univpm.it (E.L.)

**Keywords:** micronutrients, IBD, children, iron, zinc, vitamin B12, folate, vitamin D, vitamin C, vitamin E

## Abstract

Inflammatory bowel disease (IBDs), including Crohn’s disease (CD), and ulcerative colitis (UC) are complex diseases with a multifactorial etiology, associated with genetic, dietetic, and other environmental risk factors. Children with IBD are at increased risk for nutritional inadequacies, resulting from decreased oral intake, restrictive dietary patterns, malabsorption, enhanced nutrient loss, surgery, and medications. Follow-up of IBD children should routinely include evaluation of specific nutritional deficits and dietetic and/or supplementation strategies should be implemented in case deficiencies are detected. This narrative review focuses on the prevalence, risk factors, detection strategy, and management of micronutrient deficiencies in pediatric IBD.

## 1. Introduction

Inflammatory bowel diseases (IBDs), including the most common subtypes, Crohn’s disease (CD) and ulcerative colitis (UC), have become a global public health concern [1].

IBD patients present significant gastrointestinal symptoms such as diarrhea, abdominal pain, and bleeding, along with extra-intestinal manifestations, including micronutrient malabsorption and weight loss [2,3].

Although the etiology of IBD remains not completely defined, it is believed that its pathogenesis results from a complex interaction among genetic factors, immune dysregulation, and environmental triggers [4,5]. The increasing global occurrence of IBD has been associated with the Western dietary pattern [6], characterized by elevated intake of red meat and animal-based proteins, saturated fatty acids, refined sugars, and ultra-processed foods [7,8]; this epidemiological trend is expected to reach 1% of the global population within the next few years [9].

In contrast to many chronic conditions that manifest later in life, IBD often develops during adolescence or early adulthood. Approximately 10–25% of cases emerge in childhood or adolescence, with evidence suggesting a rising incidence in these age groups [10,11]. In Europe, the incidence of pediatric IBD is currently 15.3 per 100,000 person-years for Crohn’s disease (CD) and 14.8 per 100,000 person-years for ulcerative colitis (UC) [12].

Nutritional assessment and dietary management play a crucial role in the care of subjects with IBD, with particular relevance in IBD children [13,14]. The onset of IBD in childhood can adversely influence growth and development, as a result of several factors, including unbalanced and selective diet, anorexia, inflammation, malabsorption, and disease treatments, which ultimately contribute to malnutrition and impaired growth. The presence of malnutrition negatively impacts many IBD-related outcomes, such as puberty and final growth [15], treatment response [16], risks of hospitalization [17,18], and quality of life [19,20].

Pediatric patients with active IBD are at significant risk not only for energy and protein malnutrition but also for specific micronutrient deficiencies (vitamins, minerals, and trace elements). These nutritional deficits may result from multiple mechanisms, such as inadequate oral intake, intestinal malabsorption, increased gastrointestinal losses, chronic systemic inflammation, hypermetabolic state, and adverse effects associated with medications [21,22,23,24]. Inflammatory mediators can directly impair the absorption and utilization of specific nutrients, most notably iron and vitamin D [25,26].

Prominent scientific societies such as the European Society for Clinical Nutrition and Metabolism (ESPEN) and the European Society of Pediatric Gastroenterology, Hepatology and Nutrition (ESPGHAN) recommend systematic nutritional monitoring in all patients with IBD, both adults and children, with particular emphasis on periodic assessment of micronutrient status [27,28].

The micronutrients and vitamins most frequently affected in IBD include iron, selenium, zinc, vitamin D, vitamin B12, folic acid, and vitamins A, E, and C. Deficiencies in these micronutrients and vitamins may be attributed to IBD itself but can also be associated with concomitant autoimmune conditions; for example, autoimmune chronic atrophic gastritis may compromise the absorption of iron and vitamin B12, whereas celiac disease is frequently associated with impaired absorption of iron and folic acid [29].

While the role and the consequence of deficiencies of many micronutrients in IBD are well defined (such as iron and vitamin D), it remains unclear whether the reduced serum levels of other micronutrients are a cause or a consequence of disease activity, and the potential therapeutic role of supplementing many of them is not well established. Furthermore, for many micronutrients, serum determination alone does not always reflect the real bioavailability and/or storage, and other biomarkers should be considered.

This review aims to summarize the current evidence on micronutrient deficiencies in pediatric IBD, defining prevalence, risk factors, diagnostic pathways, and pitfalls, and suggesting possible treatment strategies.

## 2. Materials and Methods

Aiming to determine the prevalence, risk factors, consequences, diagnostic approaches, and therapeutic strategies for managing micronutrient deficiencies in pediatric patients with IBD, an extensive literature search was conducted through electronic bibliographic databases, including PubMed and Cochrane Library for studies published from 1984 to 2025 (last date of bibliographic databases search: 10 March 2025). The following keywords were used to include the most relevant studies: “pediatric IBD”, “micronutrient deficiency”, “diet”, and “serum micronutrients”. Eligibility criteria for articles were: English language, pediatric population (0–18 years), and edition in the last 41 years. We included the following micronutrients: iron, folic acid, zinc, selenium, and vitamins A, B12, D, E, and C.

A total of 32 articles were identified for matching the keywords and respective abstracts were read by two authors (T.G. and S.Q.). Among these, 14 articles were excluded for not matching the inclusion criteria (adult population, other languages). A summary of the 18 articles selected was made through an Excel spreadsheet and a double check by two authors (T.G. and S.Q.) was made to include the most relevant articles in the review. The most relevant findings were extracted and discussed by all the authors before manuscript preparation.

## 3. Results

### 3.1. Iron, Zinc, and Selenium

Iron is an essential trace element critical for oxygen transport, cellular respiration, immune function, and gene regulation. It is absorbed by enterocytes primarily in the duodenum and exists in two dietary forms: heme and non-heme iron. Heme iron, derived mainly from animal sources such as meat and fish, is absorbed efficiently at a rate of approximately 20–30% [30]. In contrast, non-heme iron, found in plant-based foods, has lower absorption efficiency, typically around 10% [31].

Iron deficiency (ID) and the resulting anemia may present with a range of extraintestinal manifestations, including chronic fatigue, sleep disturbances, reduced physical and cognitive performance, and impaired immune function—all of which can substantially affect the patient’s overall quality of life [32]. In the setting of IBD, the causes of ID are various, including bleeding, impaired absorption, and dietary restriction [33,34]; more recently, researchers have been focusing on other mechanisms such as ferroptosis, suggesting ferroptosis inhibitors as potential agents that could reduce inflammation in IBD [35,36,37].

Iron status is generally assessed by the evaluation of serum markers such as serum ferritin (SF), transferrin saturation (TS), and soluble transferrin receptors (sTfR). Iron deficiency anemia (IDA) is the result of iron depletion that leads to a reduced level of hemoglobin (Hb), with a consequent reduced oxygen transport to the body’s tissues. According to the WHO definition, anemia in children is defined as Hb levels inferior to two standard deviations (SD) for age [38]. In IBD subjects, iron assessment and IDA definition cannot be based only on Hb and SF; since SF is an acute phase reactant, the level of inflammation should be considered. Furthermore, the erythrocyte mean corpuscular volume (MCV), generally reduced in IDA, can be normal or increased as the consequence of other contributing factors, such as vitamin B12 and folic deficiency, anemia of chronic disease (ACD), and treatment with azathioprine (when increased MCV is considered an indicator of good compliance). Pediatric guidelines suggest diagnosing IDA in the presence of anemia and different SF cutoffs, variable with the presence of active IBD disease [39].

In various studies, up to 77–90% of children show evidence of iron depletion at IBD diagnosis [40,41,42,43,44], with prevalence varying with the definitions and cutoff used for ID and IDA. A recent US cohort study suggests how a well-defined and easy-to-follow screening algorithm increases significantly rates of IDA at diagnosis of IBD [43].

During follow-up, ID occurs in variable percentages, from 33% to 88% of IBD children [44,45,46,47,48]. Dietary data, based on a food-frequency questionnaire, show that the usual diet of IBD children does not meet the recommended intakes more frequently when compared to healthy controls [49].

Given the high prevalence of anemia in patients with inflammatory bowel disease, the North American Society of Pediatric Gastroenterology (NASPGHAN) [50], the European Crohn’s and Colitis Organization (ECCO) [51], and, more recently, the Italian Society of Gastroenterology, Hepatology, and Nutrition (SIGENP) [39] have established guidelines indicating that ferritin levels of less than 30 μg/L in patients with IBD in remission, and up to 100 μg/L during flare-ups, still suggest ID and appropriate treatment is required.

Although the optimal dose in IBD has not been established, the commonly recommended dose of oral iron is similar to patients without IBD [52]. Given that a high proportion of non-absorbed ingested iron remains in the gut, oral iron supplementation is associated with gastrointestinal side effects such as nausea, vomiting, diarrhea, abdominal pain, and constipation in up to 20% of patients [53]. Nausea and abdominal discomfort generally occur 1–2 h after intake and are generally dose-related, while other gastrointestinal side effects such as constipation and diarrhea are idiosyncratic [54].

Oral treatment may be recommended in inactive disease, while intravenous (IV) supplementation should be preferred in patients with active IBD and/or moderate-to-severe anemia, or mild-to-moderate anemia and previous intolerance to oral iron [27,39,50,51] (see Table 1).

Both low and high serum iron levels can have toxic effects on the epithelium, as a result of enhanced inflammatory responses [70]. Excessive iron supplementation may also determine epigenetic modifications, potentially influencing the condition of IBD patients [71]. While iron replacement in IBD patients can support protection against oxidative damage and repairing of the intestinal barrier, it is highly recommended to adhere to the suggested guidelines for optimal supplementation [72].

Zinc is an essential mineral that plays a key role in various aspects of cellular metabolism, including supporting the catalytic activity of numerous enzymes; regulating the immune response, inflammatory processes, protein synthesis, wound healing, and DNA synthesis; and enhancing intestinal barrier function.

Zinc deficiency is observed frequently in IBD subjects, with prevalence in pediatric studies ranging from 17 to 51% [73]. A recent meta-analysis, which also included pediatric data, estimated an overall prevalence of 50% (95% CI: 0.48–0.52) [74]. The deficiency is more pronounced at diagnosis, in Crohn’s disease [75] and in patients with high output ileostomy. Low levels of zinc may be attributed to poor absorption but also to reduced oral intake. IBD children tend to follow a low zinc diet in comparison to healthy controls, not reaching the recommended intakes [49]. Moreover, inflammation itself may be a catalyst for elevated urinary excretion of zinc [76]. Concurrently, low serum zinc levels may exacerbate inflammation by compromising the epithelial barrier, modifying mucosal immunity, and enhancing the production of pro-inflammatory cytokines.

Assessing zinc status in patients is challenging because it lacks storage mechanisms and can fluctuate significantly with intake. Furthermore, serum collection should be performed in the morning, after fasting, and include zinc-free vacuum tubes and stainless-steel needles, avoiding hemolysis, ensuring a rapid serum separation, and using anticoagulants low in zinc concentration. Serum zinc levels are considered normal between 70 to 250 μg/dL in adults, with mild deficiency manifesting when values decrease below 60 μg/dL [57]. In children, several studies have identified different normal serum ranges; however, no specific cutoffs are currently available [77,78], except for specific short age ranges (i.e., school-age children 6–9 years, as reported by Alves CX and colleagues [79]. Other markers such as urinary or hair zinc levels do not reflect acute changes in zinc bioavailability [80].

Data from adult studies [81] suggests a correlation between zinc deficiency and adverse clinical outcomes in IBD, including an increased risk of surgery, hospitalization, and complications. The same study also demonstrated that these outcomes improved with the normalization of zinc levels, suggesting that close monitoring and zinc replacement may be necessary for IBD patients, as needed [81]. Limited studies in adults showed that daily zinc supplementation in CD improves intestinal permeability [82], and improves the Crohn’s disease activity index (CDAI) score and serum zinc levels [83]. Furthermore, a randomized, placebo-controlled trial demonstrated that the administration of zinc–carnosine chelate compound enemas in patients with active UC undergoing induction therapy resulted in improved clinical responses and higher rates of remission compared to the placebo group [84]. In children, data are controversial. Interestingly, low serum zinc levels persist in a high percentage of IBD children after a high oral zinc intake [85], suggesting the need for more aggressive oral supplementation.

Selenium is another micronutrient actively involved in oxidative stress homeostasis, being a component of glutathione peroxidase and a scavenger of hydroperoxides. Selenium deficiency can result in muscle pain, weakness, cardiomyopathy, encephalopathy, lightening of hair and skin color, and anemia [86]. Different selenoproteins are involved in the inflammatory pathway of IBD, mainly selenoproteins S and K, and selenium may downregulate inflammatory signaling and IBD-relevant cytokine production [87,88] Reasons for low selenium include small intestinal involvement [89] (the main site of absorption) and reduced intake. Selenium deficiency is generally assessed by determining serum selenium levels, with both national and international guidelines offering reference ranges; for instance, the U.S. Institute of Medicine recommends a reference range of 70 to 155 μg/L [59]. Another method of evaluation is assessing the activity of selenium-dependent enzymes, but this is not routinely applicable. Data from adult studies indicate that up to 30% of IBDs have selenium deficiency [90,91]. Pediatric data are more reassuring, demonstrating normal serum values in the vast majority of IBD children. A recent Japanese study identified selenium deficiency in 10.2% of IBD with a higher prevalence in observed CD (15.3%) compared to UC (5.9%) [75].

### 3.2. Folic Acid (Vitamin B9) and Vitamin B12

These two nutrients are particularly recognized for their roles in erythropoiesis and their association with IBD-related anemia. Vitamin B12 (cobalamin) and folate (Vitamin B9) are essential for nucleic acid synthesis and red blood cell production. Erythroblasts require both nutrients for their proliferation during differentiation, and a deficiency in either leads to macrocytosis, erythroblast apoptosis, and anemia. Furthermore, folate acts as a cofactor in DNA synthesis.

In IBDs, the primary causes of folate deficiency are malabsorption, particularly in extensive CD with proximal small bowel involvement or short bowel syndrome, and inadequate intake unable to compensate for gastrointestinal losses. Other causes include medications used to treat IBD, such as methotrexate, azathioprine, and sulfasalazine, which inhibit folate absorption [92]. For IBD patients with evidence of folate deficiency, celiac disease should be always ruled out, considering the increased risk in developing celiac disease in children with IBD, recently defined as HR (hazard ratio) of 2.77 (95% CI: 1.92–4.00) [93]. Diagnostic confirmation of celiac disease, following the European (ESPGHAN) pediatric guidelines, can rely on serology when serum anti-tissue transglutaminase antibodies are above 10 times the ULN (upper limit of normal), and serum IgA is normal for age. A duodenal biopsy and histological confirmation are still required when serology is not conclusive, or subjects are IgA deficient [94]. Many of the foods richest in folate, such as legumes and leafy greens, are frequently avoided by IBD subjects, with possible nutritional deficiencies. In some countries, specific food fortification programs have markedly reduced the prevalence of folate deficiency in the general population, with particular relevancy in deficiency associated with pregnancy.

Folate deficiency is the second most common micronutrient deficiency in patients with IBD, affecting up to 30% of adult patients with CD and 10% of those with UC [95]. Pediatric data are more heterogeneous, showing deficiency in 2–40% of IBD children [40,48,95,96].

The primary clinical manifestation of folate deficiency is megaloblastic anemia. Additionally, reduced folate levels are often associated with elevated homocysteine concentrations (hyperhomocysteinemia), which may increase the risk of thrombotic events [97] in the long-term and IBD-associated cancer. Folate status can be precisely assessed through the determination of red blood cell (RBC) folate [98,99]; however, it is routinely determined by evaluating serum folate levels. Serum concentrations below 2 ng/mL is considered indicative of deficiency, while levels between 2 and 4 ng/mL require further confirmation through the measurement of methylmalonic acid (MMA) and homocysteine levels, generally normal and increased, respectively [60]. In cases of folate deficiency, in addition to dietary advice, a daily supplement is recommended, particularly for patients using methotrexate (Table 1).

Clinical manifestations of vitamin B12 deficiency include megaloblastic anemia manifesting with pallor and asthenia, oral ulcers, glossitis, neurological signs, and peripheral neuropathy [100]. Approximately 20% of adults with CD develop vitamin B12 deficiency, because of the active inflammation of the terminal ileum, where vitamin B12 is primarily absorbed, or after ileocecal surgery [101]. Ileal resections exceeding 30 cm in CD patients have been shown to increase the risk of deficiency, requiring treatment. While mucosal inflammation, surgery, and/or inadequate oral intake are the main causes of deficiency in these patients, autoimmune gastritis should be considered and ruled out (especially in adults with IBD) in cases of severe or unexplained deficiency, by measuring anti-parietal cell antibodies [102].

Pediatric studies, although limited to small populations, show figures similar to adults. A Brazilian study found 20% of patients with inactive CD having low serum B12 values [103]; similar results were reported by Yakut et al. describing 22.2% of patients with CD and 7.5% of patients with UC [95]. Other studies [104] suggest that low vitamin B12 levels are rarely observed in pediatric IBD at diagnosis and after limited ileocecal resection (<20 cm of terminal ileum resected).

To address vitamin B12 deficiency, supplementation with intramuscular cyanocobalamin is recommended once monthly, and more recently, sublingual formulations have also been introduced [105] (Table 1).

The recent Italian pediatric guidelines on anemia in IBD recommend an annual check of vitamin B12 and folic acid in patients with active ileal disease, prior surgery or on chronic therapy with sulfasalazine, thiopurines, and methotrexate [39]. Vitamin B12 serum levels below 200 pg/mL indicate deficiency, while levels between 200 and 300 pg/mL require further confirmation tests [61]. In the diagnostic work-up of both vitamin B12 and folate deficiency, methylmalonic acid (MMA) and homocysteine levels should be included [39]. Both serum MMA and homocysteine concentrations are elevated in vitamin B12 deficiency; however, an isolated increase in homocysteine with normal MMA levels suggests folate deficiency.

### 3.3. Vitamin A (Retinol)

Vitamin A is a fat-soluble vitamin that exists in several forms, which includes retinol, retinal, retinoic acid (RA), and carotenoids [64]. RA, a metabolite of retinol, plays an essential role in maintaining mucosal immune balance by supporting tolerogenic dendritic cells (DCs), regulating Th17 and T regulatory cell responses, and promoting gut homing of innate lymphoid cells. The relevance of RA deficiency in IBDs is not so clear; some data demonstrate a worse outcome in UC patients but not in CD [106].

Retinol concentrations in the serum are commonly used to assess vitamin A deficiency. In IBD adults, the prevalence of this deficiency is between 29% and 32% [107,108]. In IBD children the prevalence is lower, with different prospective and retrospective studies showing prevalence between 14 and 16% [109,110], but increased in CD children with active disease (40%) [103].

The serum concentration of RA is influenced by different factors, such as retinol-binding protein synthesis in the liver, nutritional status, and the levels of other micronutrients (i.e., zinc and carotenoid). Also, the absorption is affected by dietary factors (i.e., zinc and protein deficiencies), which may be depleted in IBD patients [110].

Dietary intake of vitamin A in IBD children is often below the recommended daily allowance (RDA). Hartman C. et al. reported poor vitamin A intake (72% of the RDA) in 39% of a cohort of 68 IBD children and adolescents [48].

While no correlation was found between vitamin A status and ileal disease, ileal resection, disease duration, or C-reactive protein (CRP) levels, CD patients with vitamin A deficiency had significantly lower body mass index and body fat compared to those with normal levels [107].

Despite this, studies involving vitamin A or RA supplementation in human populations have yielded disappointing results [111]. The efficacy of supplementation is related to some enzymatic mechanisms, and it is generally reduced if the expression of ALDH1a2 (Aldehyde Dehydrogenase 1 Family Member A2) is low and the activity of the RA-catabolizing enzyme CYP26A1 is increased [112].

### 3.4. Vitamin C

Vitamin C, also referred to L-ascorbic acid, is a water-soluble antioxidant micronutrient compound primarily absorbed in the small distal intestine. It plays a crucial role in the synthesis of collagen, carnitine, and neurotransmitters [113] and is essential in all stages of wound healing [25].

Clinical vitamin C deficiency, or scurvy, is no longer considered a historical condition of the 18th century, as its incidence has been rising in developed countries in recent decades [114]. In the general population, risk factors for clinical vitamin C deficiency include frequent dieting, anorexia nervosa, alcohol dependence, psychiatric disorders, kidney failure, and chronic gastrointestinal conditions such as CD and celiac disease [115].

Vitamin C deficiency in inflammatory bowel disease (IBD) remains poorly understood, with limited reports in recent decades. A retrospective study of 301 IBD patients estimated the prevalence of vitamin C deficiency (serum vitamin C level < 11.4 µmol/L) to be 21.6%, including 24.4% in CD patients and 16.0% in UC patients [116]. In the pediatric IBD population, vitamin C deficiency has been explored in small sample sizes with most of the studies reporting plasma ascorbic acid concentrations within the normal range [73].

Vitamin C deficiency in IBD patients are likely multifactorial, with potential risk factors including limited consumption of fruits and vegetables, surgical resection, and disease activity. Although not routinely recommended, many IBD patients follow low-residue diets to reduce intestinal motility and bacterial fermentation of fiber. Unfortunately, these diets frequently exclude fresh fruit and vegetables, which are the primary dietary sources of vitamin C [65,117]. Inadequate dietary intake is often accompanied by impaired intestinal absorption of vitamin C in these patients.

Dietary intake of vitamin C in IBD children is often lacking [48], especially compared to healthy peers. In a cohort of 110 IBD Italian children, the vitamin C/RDA ratio (%) compared to controls was significantly lower (respectively, ~75% vs. ~100%, *p* < 0.001) [49].

While repletion of vitamin C in deficient patients is essential, long-term management should emphasize the maintenance of adequate and sustained dietary levels, especially guaranteeing adequate daily consumption of fruits and vegetables (Table 1).

Unlike the relapsing and often refractory nature of IBD in many patients, vitamin C supplementation can lead to rapid resolution of symptoms, including some incorrectly ascribed to IBD. Even in IBD patients with unmeasured vitamin C levels, empiric supplementation is not unreasonable, given vitamin C’s role as an antioxidant, preventing free radical damage and reducing extracellular oxidants [118].

### 3.5. Vitamin E

Natural vitamin E includes a group of fat-soluble vitamins, among which are tocopherols and tocotrienols [119]; tocotrienols are more hydrophobic in lipid bilayer than tocopherols [120] and both tocopherols and tocotrienols can be classified as one of four isomers: α, β, γ, and δ. The dietary sources of tocopherols are plant-based oils [66,67].

In the cohort of Hartman C. et al., compared to RDA, the intake of patients with IBD was significantly poor for vitamin E (57%, *p* < 0.05) [48]. Also, in a sample of 110 Italian IBD children, the vitamin E/RDA ratio was below 50% [49].

Oxidative stress is a very common status in IBD patients [121], and the inhibition of lipid peroxidation or the scavenger of reactive oxygen species (ROS) may be an efficient strategy for prevention and treatment in these patients. Vitamin E is a strong antioxidant and acts in the neutralization of free radicals, relieving from oxidative stress [122]. Demonstrated effects of vitamin E are reported in several clinical trials, in which emerged its role in lowering oxidative stress markers, such as malondialdehyde (MDA) [123]. Moreover, in animal models, vitamin E showed the capacity of reducing intestinal inflammation with its role in the inhibition of inflammation, the protection of tight-junction proteins, and regulation of the intestinal microbiota. Also, the combination with other antioxidant molecules (i.e., selenium) resulted in a synergistic effect, useful in the prevention of experimental colitis [124,125,126].

Vitamin E deficiency is very common, particularly when the body is unable to absorb enough fat. The definition of vitamin E deficiency has not been clearly established, but plasma α-tocopherol concentration can be used as a reliable value of vitamin E status, with plasma vitamin E levels reflecting the body’s vitamin E stores, as proposed by the Institute of Medicine’s Food and Nutrition Committee. Levels of alpha-tocopherol less than 5 μg/mL are indicative of deficiency; a definitive diagnosis should be considered when the ratio between serum alpha-tocopherol and serum lipids is low [127].

The prevalence of vitamin E deficiency in pediatric IBD samples was approximately 5% [109]. A Brazilian study on nutritional changes in adolescents with CD showed significantly higher percentages of serum micronutrient deficiencies in patients compared to controls; in particular, vitamin E deficiency was the most common one. This could be evocative of a higher risk of vitamin E deficiency in CD patients. Rempel et al. found 9% (5% in CD and 4% in UC patients) low vitamin E levels in their prospective cohort study of 165 IBD subjects [42]. Similarly, 6.2% prevalence in vitamin E deficiency was observed in a cohort of 97 IBD patients at Children’s Hospital Boston [110]. This research demonstrated susceptibility for vitamin E deficiency both in CD and in UC patients, although the prevalence may differ. Furthermore, some observational studies showed an inverse relationship between vitamin E levels and the risk in developing UC [128].

Several reasons for vitamin E deficiency in IBD have been suggested. First, diet is often unbalanced because of symptoms (i.e., abdominal pain), systemic inflammation, hypermetabolism, and drug adverse effects. Secondly, intestinal absorption may be compromised, leading to fat and consequent malabsorption of liposoluble vitamins. Finally, excessive intestinal loss, effects of certain medications, or use of artificial nutrition without appropriate supplementation may contribute to a negative balance in vitamin E [129,130].

Some studies also suggested that vitamin E deficiency in IBD subjects is frequently associated with vitamin A deficiency, showing a significant and linear correlation of vitamin E levels with vitamin A levels [110]. In IBD patients, increased oxidative stress is common, and the inflammatory status compromises the fat-soluble vitamin absorption, leading to a decrease in vitamin levels, especially for vitamins A and E [130]. This suggests a synergistic role of these two vitamins in IBD-related malabsorption or dietary imbalances.

### 3.6. Vitamin D

Vitamin D is widely recognized as the primary regulator in the metabolism of calcium (Ca) and phosphorus (P), which is crucial for bone and muscle health. Recently, a growing interest in new roles of vitamin D emerged in the contexts of immune response and immunomodulation [131,132] and intestinal immunity, as well as promoting the maintenance of gut epithelial integrity [133,134,135,136].

Many studies underlined the effects of vitamin D in the pathogenesis of different diseases, such as endocrine, metabolic, cardiovascular, and gastrointestinal diseases; cancer; IBDs; and autoimmune diseases [137,138] (i.e., systemic lupus erythematosus, multiple sclerosis). Vitamin D has shown several roles in mechanisms of innate immunity (i.e., stimulation of antimicrobial peptide production, macrophage activation, and sensing the gut microbiota through Paneth cell function) [135]. An adequate range in serum vitamin D levels was not always unanimously established in all studies. Nevertheless, a value less than 20 ng/mL (or 50 mmol/L) is generally defined as vitamin D deficiency, while levels between 21 and 29 ng/mL generally indicate “insufficiency”.

Vitamin D deficiency and insufficiency in pediatric IBD patients are quite common. The prevalence of vitamin D deficiency in IBD children was between 19% and 43%, with minimal differences between cases and controls in the case-control studies [73]. Considering the prevalence according to the IBD type, a recent longitudinal population-cohort study in 165 IBD children and adolescents reported prevalence at diagnosis of 16% and 28% in CD and UC, respectively [34]. A retrospective study on 359 IBD children and adolescents reported a prevalence of 39% and 49% in CD and UC, respectively [104].

Vitamin D level is influenced by several determinants, such as latitude and sun exposure, skin pigmentation, and dietary intake (especially consumption of dairy products and vitamin D-added foods). In IBD patients, immunosuppressive treatments, cumulative use of corticosteroids, dietary restrictions, and compromised absorption [139,140] may impair the serum level of vitamin D. It is still controversial if vitamin D status is influenced by disease phenotype, disease location, and disease severity. Some authors did not show any correlation between vitamin D concentrations and the severity of the disease [141,142,143], while others reported a negative correlation with both the severity and length of the disease [144].

The most common complications of vitamin D deficiency in IBD children are malnutrition and delays in growth and puberty, but may also be one of the first symptoms of the disease [142]. Moreover, the diet of IBD patients may lack sufficient dairy products (due to common lactose intolerance) or other vitamin D-rich foods, often because of typical disease symptoms (such as diarrhea and abdominal pain) that may occur afterward [145,146,147,148]. Furthermore, Ananthakrishnan et al. have reported that a lack of vitamin D may increase the risk of malignancies in IBD patients [149].

Both children and adolescents are at high risk of vitamin D deficiency during the IBD clinical course and should always be assessed for it. Therefore, current guidelines recommend that all children with CD, who are treated with steroids, have their serum calcium and 25(OH) vitamin D levels monitored, and supplemented, if necessary, to help prevent low bone mineral density. If this occurs, osteopenia and osteoporosis should be managed according to the current osteoporosis guidelines [150]. According to the latest ESPGHAN guidelines, vitamin D supplementation is recommended in all IBD pediatric patients with serum levels below 20 ng/mL and the dosage should be weight-based [27].

## 4. Discussion and Conclusions

Inflammatory bowel diseases (IBDs) are chronic conditions with multifactorial pathogenesis, characterized by immune dysregulation, inflammation, and gastrointestinal symptoms. Many studies in the literature showed that pediatric IBD patients are at higher risk for micronutrient (vitamins, minerals, and trace elements) deficiencies, and these nutritional defects may affect growth and development, also reducing quality of life.

Our updated review aimed to identify the main characteristics of micronutrient deficiencies in pediatric IBD, especially to define the prevalence, monitoring procedures, risk factors, and clinical consequences, and to suggest adequate treatment approaches.

We confirmed that ID is the most common nutritional deficit in pediatric IBD, particularly at disease onset. Symptoms of ID often overlap with those of IBD; therefore, assessment cannot be only clinically based. The use of a standardized diagnostic algorithm, considering multiple biomarkers and accounting for the level of inflammation and disease activity, excluding other nutritional deficiencies, allows a correct diagnostic and therapeutic approach for ID in IBD children, avoiding misdiagnosis or overtreatment. Specific guidelines on the management of anemia in IBD children are currently available [39,50]. The main factors that may impact anemia include insufficient oral intake or supplementation and poor disease control. Regarding dietary intake, it is important to stress the regular consumption of iron-rich foods (i.e., meat, poultry, fish, and seafood) and/or the enhancement of iron absorption (for example, adding foods rich in ascorbic acid in the same meal with iron-rich foods). After optimizing diet, or in cases of severe IDA, treatment is recommended. While in mild disease the oral route is the first choice, in active IBD or if oral iron is not well tolerated, the IV iron formulations should be preferred (Table 1).

While selenium deficiency is rarely reported in children with IBD, zinc deficiency is frequently registered in pediatric IBD subjects, especially in newly diagnosed CD patients [73]. The lack of zinc can negatively impact growth and immune function, already compromised in IBD children. Despite this, routine evaluation is not currently recommended, but it is prudent to assess zinc status in the case of prolonged diarrhea and to supplement with oral zinc with a short course (2–4 weeks) [27] (Table 1). Other specific roles of zinc as an immunomodulator or anti-inflammatory micronutrient in pediatric IBD have not been explored extensively and require specific trials.

The prevalence of folate and vitamin B12 deficiency, although less relevant in comparison to iron and zinc, is not negligible in pediatric populations with IBD. Both these nutrients are fundamental for erythropoiesis, and deficiencies may have a significant impact on anemia. Periodic evaluations of both are suggested (Table 1), and serum evaluation is part of the extensive work-up necessary in the case of anemia in IBD, specifically when normocytic or macrocytic anemia occurs. As for prevention, diet is fundamental for reaching the recommended dietary allowance, while in the case of deficiency, specific recommendations are available (Table 1). As for the treatment of vitamin B12 deficiency, intramuscular cyanocobalamin is generally suggested; however, recently sublingual formulations have been successfully used in infants and children [151] and also seem to be a valid alternative in gastrointestinal conditions. Avoidance of intramuscular administration could successfully impact the adherence and quality of life; however, specific data on IBD patients need to be accumulated.

Vitamins A, C, and E share a potential role as antioxidants and possible implications in IBD pathogenesis, such as in many other inflammatory conditions. Oxidative stress in IBD patients is very common [152,153], and scavenging reactive species of oxygen (ROS) may be a valuable strategy for the prevention and treatment of IBD. Prevalence of these vitamin deficiencies is not irrelevant in children with IBD [48,103,109], becoming even more important in the case of concomitant fat-soluble micronutrient malabsorption (i.e., chronic liver disease or pancreatic insufficiency). Guidelines do not routinely recommend periodic serum assessment. A rational strategy could include monitoring on a clinical basis or whether other risk factors are present (severe malnutrition, concomitant cholestatic liver and/or pancreatic disease, and patients on parenteral nutrition).

The largest amount of available data concerns vitamin D deficiency. In pediatric IBD, the prevalence of vitamin D deficiency is quite heterogeneous, from 19% to 43%. Differences rely on unspecific factors such as sun exposure, latitude, skin pigmentation, and IBD-specific factors (immunosuppressive treatments, cumulative corticosteroid use, dietary restrictions, and malabsorption). The anti-inflammatory and immune-modulator properties of vitamin D suggest a role other than that of a simple nutrient [154,155]. It is still a matter of debate whether serum vitamin D levels different from those of the general population are necessary to reach these extra-skeletal effects in IBD, with some studies suggesting 30–50 ng/mL as the cutoff to be achieved [156]. However, current pediatric guidelines suggest regular oral supplementation, based on the standard (general pediatric population) cutoffs [27].

In summary, epidemiological data support the importance in regularly monitoring micronutrient levels in children with IBD, particularly iron and zinc, vitamin B12 and folate, and vitamin D. Adequate monitoring prevents the consequences of micronutrient deficiencies, particularly growth delay and impaired development, which ultimately impact the outcome of IBD. Because inadequate intake is one of the main causes of micronutrient deficiency, a regular assessment of dietary habits through validated food diaries or questionnaires collected by a specialized dietitian becomes crucial.

## 5. Future Research

While the epidemiology and clinical relevance of some nutritional deficiencies in pediatric IBD is clear (i.e., iron, vitamin B12, folate, and vitamin D), the actual impact of supplementation of these nutrients on IBD outcomes is less evident. Randomized and possibly placebo-controlled, prospective trials investigating the effect of single nutritional supplementations (particularly iron and vitamin D) on specific IBD parameters and disease activity are necessary. For other nutrients (i.e., vitamins A, C, E, and zinc) there is rationale supporting their role in the pathogenetic mechanisms of IBD, regulating immune, anti-inflammatory, and oxidative pathways. Studies exploring the effect of supplementation of these nutrients, regardless of deficiency, would add elements to the knowledge of pediatric IBD diet therapy, increasingly pointing toward the goal of a personalized dietetic strategy.

## Figures and Tables

**Table 1:** Prevalence, clinical relevance, diagnostic methods, supplementations, suggested dietary intakes of micronutrient deficiencies (iron, zinc, selenium, folate, vitamin B12, A, C, and D) in pediatric IBD.

Micronutrient	References	Clinical Relevance of Deficiency	Laboratory Parameters/Definition	Monitoring	Treatment	Recommended Intake *— Dietary Sources
Iron	[31,38,39,55,56]	Pallor, asthenia, poor growth, hair loss, sleep disorders, reduced cognitive and physical performance	Hb < 2 SD for age (WHO definition) and ↓ MCV, ↓ SF (<30 ng/mL) and ↓ TS (<20%) or SF 30–100 ng/mL in active disease	Iron status at diagnosis, every 3 months in active disease, every 6–12 months in patients in remission or mild disease	Oral (ferrous salts): 3–6 mg/kg, in inactive/mild disease IV (ferric carboxy maltose): dosage according to Ganzoni formula, # in active IBD and/or moderate-to-severe anemia or previous intolerance to oral iron	PRI: 1–6 years M-F = 7 mg/day 7–11 years M-F = 11 mg/day 12–17 years M = 11 mg/day 12–17 years F = 13 mg/day Sources: Heme-iron: meat, fish, and seafood; non-heme iron: cereals, legumes, and dark green vegetables
Zinc	[49,57,58]	Impaired growth, dermatitis, impaired vision and taste, compromised immune function	Serum zinc < 70 μg/dL	At diagnosis and annually for patients with CD. In specific situations: ileostomy, surgery, elevated intestinal losses.	Dosage not established for IBD children. A short course (2–4 weeks) of 20–40 mg of elemental zinc is generally sufficient.	PRI: 1–3 years M-F = 4.3 mg/day 4–6 years M-F = 5.5 mg/day 7–10 years M-F = 7.4 mg/day 11–14 years M-F = 10.7 mg/day 15–17 years M = 14.2 mg/day 15–17 years F = 11.9 mg/day Sources: Meat, fish, legumes, nuts. Excess of phytates or oxalates leads to zinc deficiency
Selenium	[27,59,60]	Muscle pain, weakness, pallor, cardiomiopathy	Serum selenium < 70 μg/L	Not routinely recommended	Dosage not established	AI: 1–3 years M-F = 15 μg/day 4–6 years M-F = 20 μg/day 7–10 years M-F = 35 μg/day 11–14 years M-F = 55 μg/day 15–17 years M-F = 70 μg/day Sources: plant-based foods (Brazil nuts, green vegetables, shiitake and button mushrooms, and various kinds of seeds)
Folate	[39,60]	Pallor, increased risk of osteoporosis and thrombosis (adults); possible impact on GI inflammation of IBD and growth.	↓ Hb, ↑ MCV, ↓ serum folate (<2 ng/mL) and RBC folate ↑ homocysteine	Annually in cases with active ileal disease, prior surgery, or on chronic therapy with sulfasalazine, thiopurines, or methotrexate. In every case of macrocytic anemia.	Dosage not established: a daily dose of 1 mg or a weekly dose of 5 mg appears to be sufficient. 5 mg/week orally in all children on MTX treatment.	PRI: 1–3 years M-F = 120 μg DFE/day 4–6 years M-F = 110 μg DFE/day 7–10 years M-F = 200 μg DFE/day 11–14 years M-F = 270 μg DFE/day 15–17 years M-F = 330 μg DFE/day Sources: Lentils, beans, vegetables, leafy greens, and citrus fruits
Vitamin B12	[39,61]	Pallor, glossitis, oral ulcers, neurological symptoms	↓ Hb, ↑ MCV, ↓ vitamin B12 (<200 pg/mL), ↑ MMA and homocysteine	Annually in cases with active ileal disease, prior surgery, UC with ileal pouch or anastomosis, patients on chronic therapy with sulfasalazine, thiopurines, or methotrexate. In every case of macrocytic anemia.	IM B12 therapy in patients with macrocytic anemia without clinical involvement: 250–1000 μg three times a week for 2 weeks, followed by 250 μg weekly until blood count is normal, and then 1 μg every 3 months	AI: 1–6 years M-F = 1.5 μg/day 7–10 years M-F = 2.5 μg/day 11–14 years M-F = 3.5 μg/day 15–17 years M-F = 4 μg/day Sources: Fish, tuna, shellfish, beef, liver, poultry, eggs, and dairy products.
Vitamin A	[62,63,64]	Night blindness and xerophtalmia, increased frequency of infections, and development of xeroderma and phrynoderma	Serum vitamin A (retinol) < 20 μg/dL	Not routinely recommended in the absence of chronic liver disease	Not established nor recommended supplementation, except for chronic liver disease	PRI: 1–3 years M-F = 250 μg RE/day 4–6 years M-F = 300 μg RE/day 7–10 years M-F = 400 μg RE/day 11–14 years M-F = 600 μg RE/day 15–17 years M = 750 μg RE/day 15–17 years F = 650 μg RE/day Sources: dark leafy greens, orange-colored vegetables, milk products, liver, and fish.
Vitamin C	[57,65]	Corkscrew hairs, perifollicular hemorrhages, gingival bleeding, fractures, bone reabsorption areas	Serum vitamin C < 0.2 mg/dL	Not routinely recommended	No specific recommendations for IBD 300 mg/day in case of deficiency	PRI: 1–3 years M-F = 15 mg/day 4–6 years M-F = 30 mg/day 7–10 years M-F = 45 mg/day 11–14 years M-F = 70 mg/day 15–17 years M = 100 mg/day 15–17 years F = 90 mg/day Sources: fruits and vegetables (citrus fruits, potatoes, spinach, broccoli, red peppers, strawberries, and tomatoes)
Vitamin E	[66,67]	Neurological and ocular symptoms (hyporeflexia, decreased night vision, loss/decreased vibratory sense; however, limb and truncal ataxia, muscle weakness) and cardiac arrhythmias. In IBD: increased oxidative stress and consequent inflammation.	Serum vitamin E < 5 mg/L	Not routinely recommended in the absence of chronic liver disease	Not established nor recommended supplementation, except for chronic liver disease	AI: 1–2 years M-F = 6 mg/day 3–9 years M-F = 9 mg/day 10–17 years M = 13 mg/day 10–17 years F = 11 mg/day Sources: Tocopherols: vegetal oils (soybean, corn olive, canola flaxseed, walnut); Tocotrienols: palm and rice bran oil and grains (wheat germ, oats, rice, and corn)
Vitamin D	[27,68,69]	Malnutrition and delays in growth and puberty; fractures and reduced bone density	Serum vitamin D < 20 ng/mL (or 50 nmol/L)	Routinely recommended in all patients with IBD, at diagnosis and follow-ups	Vitamin D supplementation in IBD children with deficiency. Dosage: standard weight-based dose. High doses (i.e., ≥2000 IU daily or 50,000 IU weekly) and long-term treatment may be necessary to maintain sufficiency.	AI: 1–17 years M-F = 15 μg/day Sources: fatty fish livers, dairy products, fortified food

CD = Crohn disease, Hb = hemoglobin, SD = standard deviations, MCV = mean corpuscular volume, IDA = iron deficiency anemia, SF = serum ferritin, TS = transferrin saturation, IV = intravenous, PRI = population reference intake, M = males, F = females, IBD = inflammatory bowel disease, AI = adequate intake, MMA = methylmalonic acid, UC = ulcerative colitis, IM = intramuscular, GI = gastrointestinal, RBC =red blood cells, MTX = methotrexate, DFE = dietary folate equivalent. RE = retinol equivalent. * *Recommended intake referring to European Food Safety Authority* (EFSA). PRI = population reference intake (intake of a nutrient that is likely to meet the needs of almost all healthy people in a population), AI = adequate intake (AI) is a dietary recommendation used when there are too few data to calculate an average requirement. An adequate intake represents the average nutrient level consumed daily by a typical healthy population, which is assumed to be sufficient for the needs of that population. # Ganzoni formula = total iron deficit [mg] = body weight [kg] × (target Hb-actual Hb) [g/dL] × 2.4 + depot iron [mg]. The factor 2.4 is derived from the following assumptions: (a) Blood volume 70 mL/kg of body weight ~7% of body weight; (b) Iron content of hemoglobin 0.34%. Factor 2.4 = 0.0034 × 0.07 × 10,000 (conversion for g/dL). Iron stores are calculated using 15 mg/kg if the body weight is below 35 kg and 500 mg if the body weight is above 35 kg.

## Data Availability

No new data were created for this article. Results of the literature research collected by the authors are presented in the article.

## Abbreviations

IBD	Inflammatory Bowel Disease
CD	Crohn’s Disease
UC	Ulcerative Colitis
ESPEN	European Society of Parenteral and Enteral Nutrition
ECCO	European Crohn’s and Colitis Organization
NASPGHAN	North American Society of Pediatric Gastroenterology
SIGENP	Italian Society of Gastroenterology, Hepatology, and Nutrition
ID	Iron Deficiency
IDA	Iron Deficiency Anemia
MMA	Methylmalonic Acid
RA	Retinoic Acid
RDA	Recommended Dietary Allowances
